# Comparative outcomes of Hugo RAS and Versius surgical systems versus da Vinci in four urological procedures: a systematic review

**DOI:** 10.1007/s11701-026-04125-7

**Published:** 2026-11-06

**Authors:** Sarujan Thayalan, Saif Toubasey, Cynthia O’Sullivan, Ben Challacombe

**Affiliations:** 1 Faculty of Life Sciences and Medicine, King’s College London, London, UK; 2 Imperial College School of Medicine, Imperial College London, London, UK; 3 Department of Urology, Guy’s and St Thomas’ NHS Foundation Trust, London, UK

**Keywords:** Systematic review, Urology, Robotic surgery, da Vinci, Hugo RAS, Versius

## Abstract

Robot-assisted surgery has transformed urological surgery, with the da Vinci Surgical System dominating for over two decades. Following the expiry of key Intuitive patents in 2019, two particular platforms - Medtronic’s Hugo™ RAS system and CMR Surgical’s Versius^®^ Surgical Robotic System - have gained regulatory approval and entered urological practice. Comparative evidence between these platforms and da Vinci^®^ across urological procedures remains limited. This systematic review follows PRISMA guidelines and a prospectively registered protocol (PROSPERO: CRD420261319741). MEDLINE, Embase, CENTRAL, Scopus, and Web of Science were searched from January 2016 to August 2026. Comparative studies of Hugo™ RAS or Versius^®^ versus da Vinci^®^ in patients undergoing robot-assisted radical prostatectomy (RARP), robot-assisted partial nephrectomy (RAPN), pyeloplasty, or radical cystectomy were eligible. Risk of bias was assessed using ROBINS-I. 19 studies (21 procedural comparisons) involving 2,482 patients were included. In RARP, blood loss and length of stay showed substantially overlapping ranges between platforms, with no consistent direction of effect, though setup and docking times were consistently longer with the newer platforms. Positive surgical margin rates and early continence recovery overlapped widely in RARP. In RAPN, warm ischaemia times differed by five minutes or less across reporting studies, and trifecta rates were within 5 percentage points. The single small pyeloplasty study reported identical median operative times, but provided minimal comparative information. Early comparative evidence has not demonstrated consistent differences in peri-operative, oncological or early functional outcomes between Hugo™ RAS or Versius^®^ and da Vinci^®^ in experienced, high-volume centres. However, the included studies were small, non-randomised and not powered to detect clinically meaningful differences or to demonstrate equivalence. These findings therefore represent an absence of evidence of a difference rather than evidence of comparable effectiveness. Prospective studies including robotic-naïve centres are needed to advance our understanding.

## Introduction

Urology has historically been at the forefront of adopting new technology in surgery [1], and robotics has been no exception. Robot-assisted radical prostatectomy (RARP) is the most established example of this, now serving as the standard surgical approach for localised prostate cancer in most high-income countries, as recommended by the European Association of Urology (EAU) [2]. Robotic platforms are also increasingly being used for other urological procedures - the most common being robot-assisted partial nephrectomy (RAPN), radical cystectomy and pyeloplasty [3]. The da Vinci^®^ Surgical System (Intuitive Surgical, Sunnyvale, CA) has been at the centre of this transformation, having first received regulatory approval in 2000 and since then dominating the field of robotics for over two decades. It has demonstrated favourable peri-operative outcomes compared with both open and laparoscopic approaches, including reduced blood loss, shorter hospital stays and lower complication rates [4, 5].

However, the adoption of robotic platforms is influenced by factors beyond clinical outcomes. Intuitive Surgical held extensive patent protection over core robotic surgery technologies, with important patents expiring from 2019 onwards. Since the expiry of these patents, several novel platforms have entered the market [6, 7], aiming to increase competition in the robotic surgery market and address commonly cited limitations of existing systems. These include high cost, expensive single-use consumables and closed proprietary architecture [8]. Of these, two robotic systems in particular have gained clinical traction in urology: the Hugo™ RAS (Medtronic, Minneapolis, MN) and the Versius^®^ Surgical Robotic System (CMR Surgical, Cambridge, UK) [9, 10].

Having received CE certification in October 2021, the Hugo™ RAS has been adopted across more than 30 countries and received FDA clearance in December 2025, marking its entry into the US market [11]. In contrast to the da Vinci^®^, it consists of four independent modular carts, an open console with “pistol”-shaped hand controllers and integration with the Touch Surgery™ AI platform [12]. Versius^®^ received CE marking in 2019 and FDA authorisation in 2024, surpassing 40,000 procedures completed globally by early 2026 [13]. Its key design features include a compact, portable cart system, reusable instruments and an open console [14]. Both these systems were designed to be more accessible and cost-effective in a wider range of hospital settings and have been adopted across multiple specialties including urology, gynaecology and colorectal surgery [15].

The current published literature mainly consists of single-arm feasibility studies from high-volume centres which often look at initial experiences in small, carefully selected cohorts with short follow-up periods. While these studies are of use, they do not answer the clinically important question of how these systems compare in outcomes to the well-established da Vinci^®^ system. Several systematic reviews have addressed Hugo™ RAS or the broader group of novel multiport platforms [16–18]. These have either been confined to a single platform or procedure, or have pooled multiple novel systems into a single comparator arm, from which platform-specific estimates are largely not recoverable. The present review differs in two respects: it assesses Hugo™ RAS and Versius^®^ separately rather than combining them into a single novel-platform group, and it synthesises comparative evidence across four prespecified urological procedures using a search updated to August 2026.

We synthesise the available comparative evidence across four index urological procedures: RARP, RAPN, pyeloplasty, and radical cystectomy. These were selected as the procedures most frequently performed robotically in urology, chosen to allow platform performance to be assessed across more than one operative context. Our conclusions therefore apply to these procedures rather than to urological robotic surgery as a whole. We aimed to assess whether available data demonstrates consistent differences in peri-operative, oncological and functional outcomes between Hugo™ RAS or Versius^®^ and the da Vinci^®^ standard - a question with direct implications for healthcare systems considering investment in a new robotic platform. We also address something the outcome data alone cannot answer: why uptake of these systems has remained concentrated in a small number of high-volume centres despite regulatory approval.

## Methods

### Search strategy

This systematic review was conducted in accordance with PRISMA guidelines [19] following a prospectively registered protocol (PROSPERO: CRD420261319741). A comprehensive literature search was performed across MEDLINE, Embase, the Cochrane Central Register of Controlled Trials (CENTRAL), Web of Science Core Collection, and Scopus from 1 January 2016 to 1 August 2026. There were no language restrictions. The search strategy combined keywords and controlled vocabulary terms specific to the robotic platforms of interest and the urological procedures being studied. Boolean operators were used to combine platform and procedure specific terms. ClinicalTrials.gov and the WHO International Clinical Trials Registry Platform were also searched for ongoing or unpublished studies. The full search strategy for each database and registry is provided in Appendix A.

Retrieved records were imported into Rayyan for removal of duplicates. Screening was carried out independently by 2 reviewers with any disagreements resolved by discussion and if necessary, consultation of a third reviewer.

### Study inclusion/ exclusion criteria, and outcomes

Studies were eligible if they met all of the following criteria:

- Adult patients (age ≥ 18 years) undergoing RARP, RAPN, pyeloplasty, or radical cystectomy using either the Hugo™ RAS system or the Versius^®^ robotic surgical system.
- Contained a comparator group of adult patients undergoing the same procedure using the da Vinci^®^.
- Reported at least one peri-operative, oncological or functional outcome of interest.

Both prospective and retrospective studies were eligible for inclusion. We excluded case reports, case series without comparator groups, narrative reviews, systematic reviews, editorials and letters. Conference abstracts with extractable comparative data were included. Where multiple publications reported outcomes from the same cohort, the most complete dataset was used.

Four index procedures were specified a priori in the registered protocol: RARP, RAPN, pyeloplasty and radical cystectomy. Restricting the review to a single procedure would have limited assessment of platform performance to one operative context, whereas including every urological procedure risked diluting the synthesis with isolated reports. We therefore selected the urological procedures most frequently performed robotically, which also span oncological and reconstructive indications, allowing platform performance to be assessed across differing technical demands while retaining a realistic prospect of identifying comparative data.

Primary outcomes included intra-operative complications, post-operative complications within 90 days (Clavien-Dindo classification [20]), operative time, estimated blood loss (EBL) and positive surgical margin (PSM) rates. Functional outcomes were operation specific. Urinary continence (defined as 0–1 safety pads per day) and erectile function (self-reported return of spontaneous erections) were selected for RARP. Partial nephrectomy functional outcomes included changes in eGFR, warm ischaemia time (WIT) and trifecta achievement (negative surgical margins, no Clavien-Dindo complications ≥ 3 and decline in eGFR ≤ 30%). For the purpose of standardised comparison, functional outcomes were extracted at three months, as this was the most consistently reported postoperative timepoint across included studies – studies reporting only at other timepoints were not included in the three-month synthesis.

Outcome definitions were specified at PROSPERO registration and compared against those used in each included study during extraction. Where a study’s definition matched, data were extracted directly. Where it differed modestly, data were extracted and the discrepancy was flagged in the synthesis. Where it could not be reconciled with the specified definition (for example scale scores or different timepoints), the outcome was reported separately or omitted from the relevant synthesis.

### Data extraction

For each included study, data was extracted on study characteristics, patient demographics (age, BMI), and preoperative variables (pre-op PSA for RARP patients, tumour size and R.E.N.A.L score for RAPN patients). Peri-operative outcomes extracted included operative time, EBL, length of stay (LOS) and complication rates. For RAPN studies, WIT, and trifecta outcomes were additionally extracted where reported. Data extraction was performed by one reviewer and independently checked by a second reviewer.

### Risk of bias assessment

Risk of bias was assessed using the ROBINS-I tool [21] across seven domains: confounding, selection of participants, classification of interventions, deviations from intended interventions, missing data, measurement of outcomes and selection of the reported result. Each domain was judged as being low, moderate, serious or critical risk of bias. Risk of bias was assessed independently by 2 reviewers with any disagreements resolved by discussion and if necessary, consultation of a third reviewer. The overall risk of bias was determined by the highest level of risk in any domain. Conference abstracts were assessed with particular caution for selective reporting (D7), given the limited methodological and outcome information available in abstract-only reports.

### Statistical analysis

A structured narrative synthesis was performed, grouping studies by procedure and comparator platform. Quantitative pooling was considered for each outcome and judged inappropriate for the reasons below.

Operative time was neither consistently reported nor defined: only seven of fifteen RARP comparisons reported a total; the remainder reported setup, docking, console or anastomosis times in isolation or combination. EBL varied four-fold across comparable cohorts and was reported without any measure of dispersion in four studies. Major complications were sparse, with zero events in both arms in three of the seven RARP comparisons reporting them. Erectile function was assessed with differing instruments and timepoints, as noted in Sect.  3.4.2.

PSM and three-month continence were the outcomes most suited to pooling, being binary and reported by twelve and seven Hugo™ RAS comparisons respectively. We nonetheless judged synthesis inappropriate: thirteen of nineteen studies were at serious risk of bias on ROBINS-I, where pooling improves precision without reducing bias. Cohorts also captured differing points on the learning curve, with several studies identifying CUSUM breakpoints within their own series. For RAPN and pyeloplasty, too few comparisons were available, with marked imbalance between arms in one case (*n* = 21 versus *n* = 344).

A structured narrative synthesis was therefore considered more appropriate, preserving study-level context rather than presenting summary effects the underlying data do not support. Where included studies reported statistical comparisons between platforms, these are presented within the narrative synthesis; tabulated outcomes are presented descriptively and were not subjected to further statistical testing.

A post hoc sensitivity analysis excluding studies reported only in abstract form was performed and is presented in Sect.  3.7.

## Results

### Study selection

**Fig. 1:**
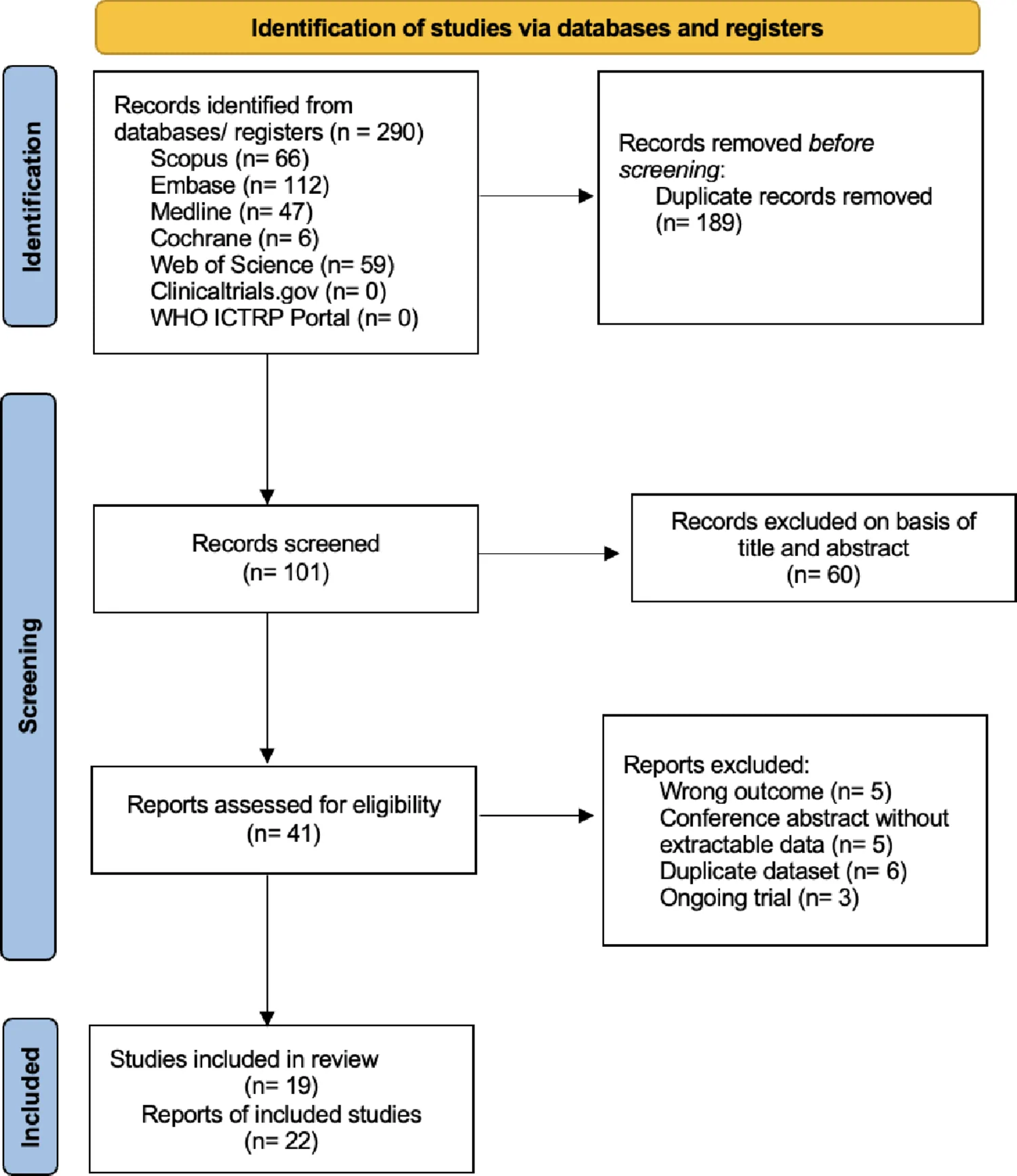
PRISMA flow diagram showing literature search results

The literature search identified 290 records across all databases. 189 duplicates were removed, leaving 101 records for title/ abstract screening. 41 reports were assessed for eligibility, with 22 reports of 19 studies meeting the inclusion criteria. These 19 studies contributed 21 procedural comparisons. The study selection process is summarised in the PRISMA flow diagram (Fig. 1).

### Study characteristics

Nineteen studies involving 2,482 patients were included, contributing 21 procedural comparisons: 15 RARPs, 5 RAPNs and 1 pyeloplasty, with no eligible cystectomy studies identified. Two studies contributed more than one comparison. Hussein et al. [22] reported outcomes for both RAPN and pyeloplasty, and the COMPAR-P trial (NCT05766163) compared the da Vinci^®^ Xi with both Hugo™ RAS and Versius^®^ in the same cohort; its da Vinci^®^ arm (*n* = 50) serves as the comparator for both comparisons and is counted once in patient totals [23–25]. One further study was reported across multiple publications: Prata et al. reported an initial matched analysis of 52 pairs in a full-text publication [26], with a subsequent conference abstract reporting an updated analysis of 105 pairs from the same two-centre cohort [27]. These were treated as a single study, with outcomes extracted from the full paper unless otherwise stated.

All studies were published between 2023 and 2026, with the majority conducted at single centres in Europe. Italy was the most represented country with six studies, followed by Spain with four, England with two, and the remaining studies were from Belgium, Denmark, Taiwan, Hong Kong, Japan, India and Pakistan. Seventeen studies compared Hugo™ RAS against da Vinci^®^ whilst three compared Versius^®^ and da Vinci^®^ (the COMPAR-P trial contributed one comparison to each, and is counted once in the study total). Eight studies were retrospective, nine were prospective studies and two were mixed comparative cohort studies. Five studies employed matching to control for baseline differences between the two groups [26, 28–31]. Of note, no eligible randomised controlled trials were identified. Key study characteristics are summarised in Table 1.

**Table 1:** Characteristics of included studies

Author	Year	Country	Comparator Platform	Procedure(s)	Sample size (Comparator/da Vinci^®^)	Matching Performed	Study Design	Study Period
Ragavan et al.	2023	India	Hugo™	RARP	17/17	Yes	Comparative cohort (mixed)	July 2021 - May 2022
Antonelli et al. (COMPAR-P)	2024–2026	Italy	Hugo™	RARP	50/50	No	Prospective comparative cohort	March 2023 onwards
Bravi et al.	2024	Belgium	Hugo™	RARP	164/ 378	No	Retrospective comparative cohort	2021–2023
Brime Menendez et al. 2024	2024	Spain	Hugo™	RARP	75/75	No	Prospective comparative cohort	March 2022 - July 2023
Gandi et al.	2024	Italy	Hugo™	RARP	99/99	Yes	Retrospective comparative cohort	September 2021 - September 2023
Olsen et al.	2024	Denmark	Hugo™	RARP	19/11	No	Prospective comparative cohort	April 2022 - November 2022
Ou et al.	2024	Taiwan	Hugo™	RARP	30/30	No	Retrospective comparative cohort	March 2023 - August 2023
Sultani Tehrani et al.	2024	England	Hugo™	RARP	33/33	Yes	Comparative cohort (mixed)	February 2023 – December 2023
Bianchi et al.	2025	Italy	Hugo™	RARP	26/26	No	Prospective comparative cohort	July 2023 – July 2024
Ko et al.	2025	Hong Kong	Hugo™	RARP	20/20	Yes	Prospective comparative cohort	January 2021 – August 2023
Morizane et al.	2025	Japan	Hugo™	RARP	16/81	No	Retrospective comparative cohort	April 2022 – April 2024
Piazza et al.	2025	Italy	Hugo™	RARP	106/203	No	Prospective comparative cohort	December 2023 – September 2024
Brime Menendez et al. 2026	2026	Spain	Hugo™	RARP	25/25	No	Prospective comparative cohort	September 2024 – September 2025
Dibitetto et al.	2024	Italy	Versius^®^	RARP	53/53	No	Retrospective comparative cohort	July 2021 – July 2023
Antonelli et al. (COMPAR-P)	2024–2026	Italy	Versius^®^	RARP	50/50	No	Prospective comparative cohort	March 2023 onwards
Hevia Palacios et al.	2023	Spain	Hugo™	RAPN	17/18	No	Prospective comparative cohort	January 2022 onwards
García Rojo et al.	2024	Spain	Hugo™	RAPN	25/25	No	Prospective comparative cohort	March 2022 – June 2023
Davies et al.	2025	England	Hugo™	RAPN	21/344	No	Retrospective comparative cohort	May 2023 – May 2025
Prata et al.	2026	Italy	Hugo™	RAPN	52/52	Yes	Retrospective comparative cohort	October 2022 – September 2025
Hussein et al.	2023	Pakistan	Versius^®^	RAPN, pyeloplasty	6/4, 9/25	No	Retrospective comparative cohort	2021–2022

The same da Vinci ^®^ comparator cohort from the COMPAR-P study contributes to both the Hugo™ RAS and Versius^®^ comparisons and is counted once in the overall patient total

### Risk of bias assessment

**Fig. 2:**
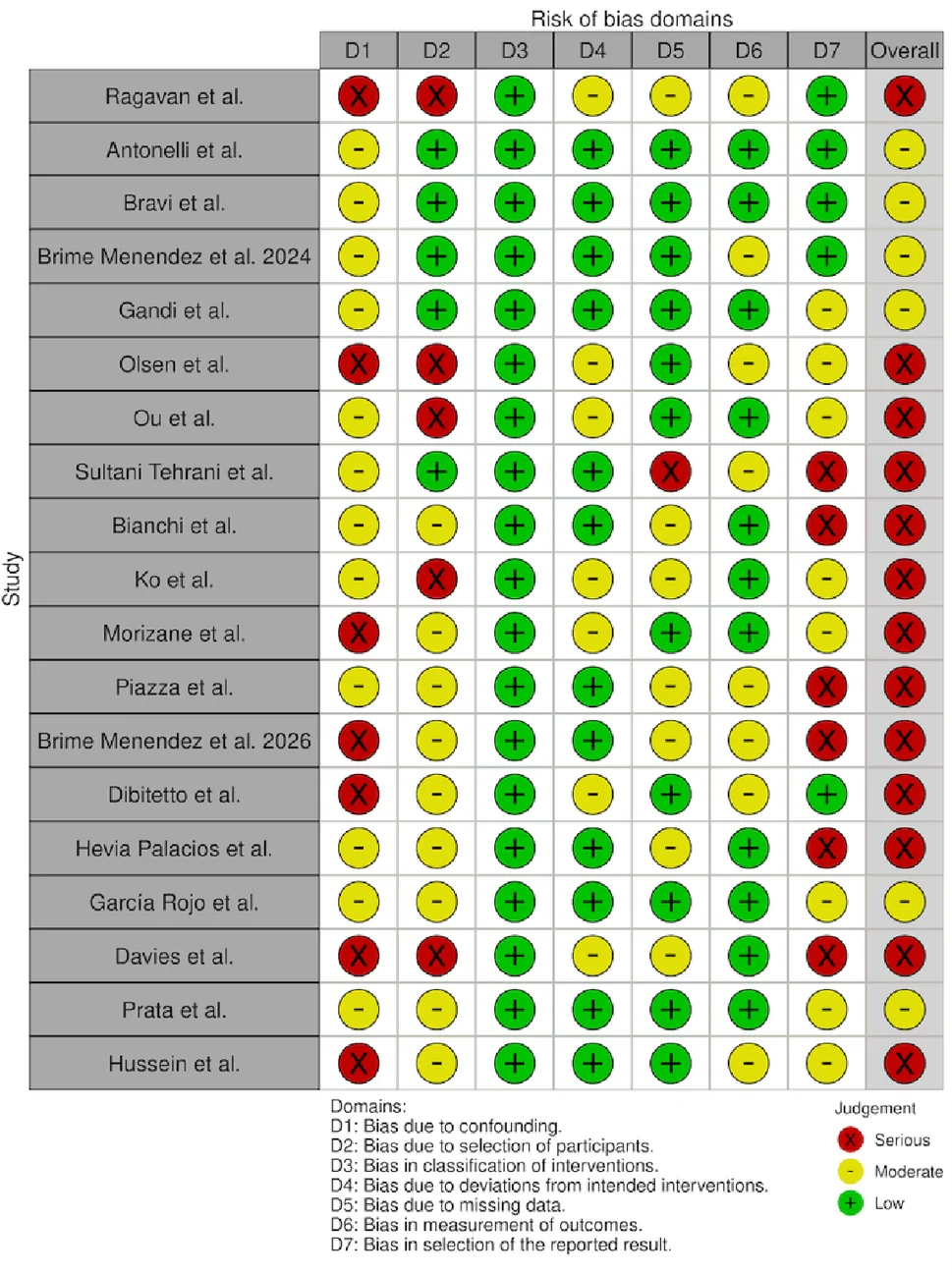
ROBINS-I risk of bias assessment traffic-light plot

**Fig. 3:**
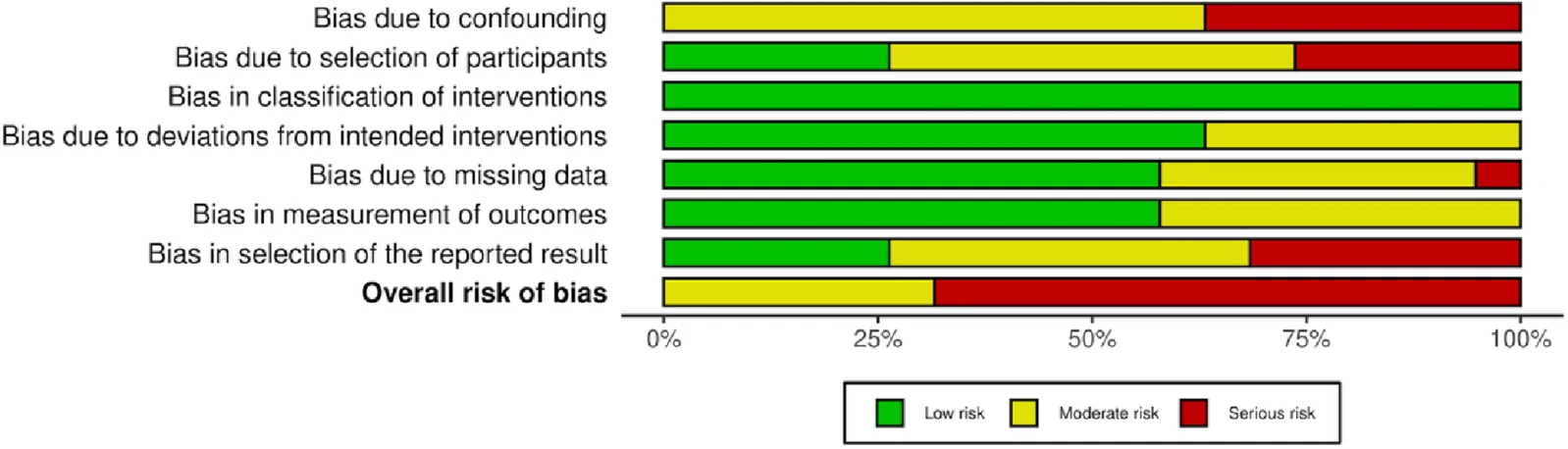
Summary of risk of bias judgements across domains

Overall, 13 of the 19 included studies were rated serious risk of bias and 6 as moderate risk on ROBINS-I, (Figs. 2 and 3). The most frequent concerns involved confounding, (D1) and participant selection, (D2), reflecting the non-randomised designs and learning curve effects of most studies. Selective reporting, (D7) and missing data, (D5) were also issues, particularly in conference abstracts and early cohort studies with limited follow-up. Only a small number of studies used patient matching methods to address confounding.

### Robot-assisted radical prostatectomy (RARP)

Fifteen comparisons reported RARP outcomes, which included thirteen Hugo™ RAS comparisons and two Versius^®^ comparisons [23, 32]. In total, 1,884 patients were included. Baseline characteristics were broadly comparable between arms across studies, as summarised in Table 2.

#### Peri-operative outcomes in RARP

Peri-operative outcomes are presented in Table 3. Setup and docking times were consistently longer with Hugo™ RAS. Antonelli et al. reported significantly longer setup and console times with Hugo™ RAS (55 vs. 37 min and 126 vs. 97 min respectively, both *p* < 0.001) [24], with CUSUM analysis identifying learning curve breakpoints at 22 and 17 cases for each phase. Bianchi et al. similarly found significantly longer docking, anastomosis and overall surgical times with Hugo™ RAS (all *p* < 0.03) [33], resolving after approximately 20 consecutive cases. Ou et al. found anastomosis time was significantly longer with Hugo™ RAS whilst overall console time remained equivalent [34]. Total operative time was reported in seven comparisons with an inconsistent direction of effect: three reported shorter times with the novel platform [30, 35, 40] and four longer [28, 32, 38, 39]. Morizane et al. paradoxically reported shorter Hugo™ RAS operative times (278.5 vs. 348 min, *p* < 0.001) [35], although the authors acknowledged that allocation of more experienced surgeons to the newer platform may have contributed to this finding. Brime Menendez et al. 2026 also reported a shorter console time with Hugo™ RAS (106 vs. 116.87 min) [36]. The COMPAR-P trial reported significantly longer setup and console phases with Versius^®^ than da Vinci^®^ (59.5 vs. 37 min and 152 vs. 97 min, both *p* < 0.001), with CUSUM analysis identifying a console-phase breakpoint at 30 cases and no learning effect for setup [23]. Dibitetto et al. also reported markedly longer operative time with Versius^®^ compared to da Vinci^®^ (170 vs. 96 min, *p* < 0.001) [32].

EBL ranged from 100 to 400 mL in novel-platform arms and 100 to 350 mL in da Vinci^®^ arms, with LOS similarly overlapping and no consistent direction of effect across studies. Major complications were infrequent, with no events in either arm in three of the seven reporting comparisons. In the remaining four they were more frequent with the novel platform (2 vs. 1, 1 vs. 0, 2 vs. 0 and 4 vs. 2 events), though the numbers are too small to support any inference. Olsen et al. reported one Clavien-Dindo grade 3b complication - a mechanical ileus attributable to trocar access closure rather than the robotic system [37]. Technical performance was a recurring difference between platforms. Piazza et al. found significantly higher intra-operative malfunction rates with Hugo™ RAS than da Vinci^®^ (14% vs. 0.5%, *p* < 0.001), with Bianchi et al. reporting similar findings [33, 38]. Antonelli et al. recorded 20 versus 4 malfunctioning events with Hugo™ RAS [24], and 38 versus 4 in the Versius^®^ arm of the same trial, with individual events consuming up to 16 min [23]. Satisfaction was lower with both novel platforms (Hugo™ 72%, 44% and 47%; Versius^®^ 43%, 43% and 39% for surgeon, assistant and scrub nurse respectively) [23, 24].

#### Functional and oncological outcomes in RARP

Functional and oncological outcomes are presented in Table 4. Urinary continence at three months ranged from 55 to 100% for novel platforms versus 50–100% for da Vinci^®^, with the upper bound at both ends driven by a single small-cohort study in which all patients in both arms used one protective pad (Ragavan et al., 17/17 patients) [30]. Among the larger comparative datasets, differences were small. Gandi et al., the only propensity score-matched study in this subgroup, reported 74.7% vs. 73.7% [28], and Bravi et al., the largest dataset (*n* = 542), reported 81% versus 79% [39]. Wider differences were confined to the smallest cohorts and ran in both directions − 76.7% vs. 53.3% favouring Hugo™ in one series [34], and 58% vs. 82% favouring da Vinci^®^ in another [37]. Most comparisons reporting three-month continence used the specified 0–1 pad threshold or an equivalent social-continence definition. Olsen et al. applied a stricter definition of complete pad-freedom [37], which yielded lower rates in both arms, and Brime Menendez et al. 2024 used a comparable threshold but reported at six months and was therefore not included in the three-month continence synthesis [40].

Erectile function was reported inconsistently, using different instruments, thresholds and follow-up timepoints. Three studies reported an outcome mappable to our binary definition at three months [31, 34, 37]. Olsen et al. reported erectile function on an ordinal scale, and patients reporting no or partial dysfunction were classified as potent [37]. Two further studies reported post-operative IIEF-5 scores without a binary potency threshold and so are reported separately. Both came from the same group and reported divergent findings: Brime Menendez et al. 2024 found near-identical mean scores at three months (8.67 ± 6.03 vs. 9.28 ± 5.30, *p* = 0.42) [40], while their 2026 series reported significantly lower scores in the Hugo™ arm (6.21 ± 3.46 vs. 11.37 ± 7.03, *p* = 0.004) [36]. All remaining studies either reported baseline IIEF only or did not report erectile function at all.

PSM rates ranged from 9 to 37. for novel platforms and 9–40% for da Vinci^®^. Direction of effect was inconsistent: of thirteen comparisons, six reported a numerically lower rate with the novel platform, five a higher rate, and two identical rates. Brime Menendez et al. 2024 was the only study to report a significantly higher PSM rate in the Hugo™ arm (20% vs. 10.67%, *p* = 0.034) [40]. The authors suggested this may reflect inadequate grasping forceps causing capsular damage during handling of the specimen, rather than a true oncological disadvantage of the platform.

### Robot-assisted partial nephrectomy (RAPN)

Five studies reported outcomes for RAPN, comprising four Hugo™ RAS comparisons and one Versius^®^ comparison [22], totalling 564 patients. Baseline characteristics were broadly comparable between arms across studies, as summarised in Table 5.

#### Peri-operative outcomes in RAPN

Peri-operative outcomes are presented in Table 6. WIT was reported in three comparisons and differed by five minutes or less in each (19 vs. 24 [47], 9.9 vs. 14.4 [41] and 19.2 vs. 17.8 min [42]), two favouring Hugo™ RAS and one da Vinci^®^. Similar to the RARP cohorts, García Rojo et al. reported shorter docking time with da Vinci^®^ (12.56 vs. 20.08 min, *p* < 0.01), though console time was similar (102.28 vs. 103.15 min, *p* = 0.86). Interestingly, renorrhaphy time was shorter with Hugo™ RAS (14.33 vs. 18.84 min, *p* = 0.024) [41]. Prata et al., the only propensity score-matched study in this group, reported longer total operative times with the Hugo™ RAS (112.5 vs. 67.5 min, *p* < 0.001) [26], though console time was identical (*p* = 0.92), suggesting the difference reflects setup and docking time rather than the surgery itself. EBL was divided between studies: it was higher in the Hugo™ arm in Prata et al. (271.3 vs. 172.5 mL, *p* = 0.001) [26], while Davies et al. reported significantly lower EBL with Hugo™ RAS (102 vs. 183 mL, *p* = 0.03) [42], though WIT was longer without reaching statistical significance. No intra-operative complications were recorded in the Hugo™ arm, compared with 47 (13.7%) in the da Vinci^®^ comparator. However, PADUA scores were significantly lower in the Davies Hugo™ cohort (7 vs. 8, *p* = 0.01), suggesting less complex tumour anatomy, and the Hugo™ arm was substantially smaller (*n* = 21 vs. 344).

Hussein et al. provided the only Versius^®^ comparison for RAPN, reporting shorter operative time for Versius^®^ (170 vs. 185 min, *p* = 0.02) [22] with no other significant differences.

#### Functional and oncological outcomes in RAPN

Oncological and functional outcomes are presented in Table 7. PSM rates ranged from 0 to 17.6% for Hugo™ RAS arms and 0-11.1% in the da Vinci^®^ arms. Three of four comparisons reported a numerically higher rate with Hugo™ RAS, though absolute differences were small (6.5, 4.0 and 5.8 percentage points) and all four Hugo™ RAS arms had fewer than 55 patients. Trifecta rates were within 5 percentage points in both reporting studies (84% vs. 88% and 89% vs. 90%) [26, 41]. Both studies reporting trifecta applied closely similar criteria, with minor differences: Prata et al. specified no Clavien-Dindo ≥ 3 complications with eGFR reduction assessed at discharge, while García Rojo et al. specified no Clavien-Dindo > 3 complications with postoperative eGFR at an unspecified timepoint. These rates are therefore comparable but not strictly equivalent. Prata et al. reported a higher eGFR at discharge in the Hugo™ RAS group (87.3 vs. 74.5 mL/min/1.73 m², *p* = 0.03) [26], though the authors attributed this to a longer observation period. An updated analysis of the same cohort, reported in abstract form, expanded the matched sample to 105 pairs following learning-curve completion, reporting trifecta achievement of 80.0% versus 89.5% (*p* = 0.055) and Clavien-Dindo ≥ 2 complications of 10.5% versus 1.0% (*p* = 0.071) [27]. No study reported recurrence or longer-term oncological outcomes.

### Other procedures

One eligible pyeloplasty study was identified from Hussein et al., comparing 9 Versius^®^ and 25 da Vinci^®^ cases. Median operative time was identical (Versius^®^ 130 [103–173] vs. da Vinci^®^ 130 [110–150] min). EBL (100 [50–100] vs. 50 [40–100] mL) and LOS (3 [2–3] vs. 2 [1–4] days) were numerically higher with Versius^®^, with overlapping interquartile ranges [22]. No statistically significant differences were identified. There were no conversions to open surgery or malfunctions. No functional outcome data were reported. As with the RAPN findings from this study, the small sample size provides limited comparative evidence.

No eligible Hugo™ RAS pyeloplasty comparative studies were found. No studies were identified for either Hugo™ RAS or Versius^®^ radical cystectomy, representing crucial gaps in the current evidence base.

### Sensitivity analysis

A post hoc sensitivity analysis was performed excluding the six studies reported only in abstract form - four RARP comparisons [29, 33, 36, 38] and two RAPN comparisons [42, 47] - all of which compared Hugo™ RAS with da Vinci^®^, leaving the Versius^®^ evidence base unaffected. This left thirteen studies contributing fifteen procedural comparisons.

The principal findings were unchanged. Setup and docking times remained consistently longer with Hugo™ RAS. For EBL, LOS, PSM and three-month continence, the observed ranges continued to overlap, with no consistent direction of effect.

Abstracts contributed detail rather than direction. Evidence for higher intra-operative device malfunction with Hugo™ RAS rested substantially on abstract reports, leaving Antonelli et al. as the principal quantitative source [24], and the reported PSM ranges narrowed in both RARP and RAPN as the extreme values were lost. The effect was most marked in RAPN, where only two Hugo™ comparisons remained [26, 41], and in erectile function, where excluding Brime Menendez et al. 2026 left no study reporting a significant difference between platforms [36]. Conversely, several of the differences that did persist, (longer setup and docking times), were those originally identified in full publications.

All six abstracts had been rated at serious risk of bias for selective reporting (D7), and were the only studies to receive that rating in this domain. Their exclusion therefore removed every serious D7 judgement from the review. Risk of bias judgements in other domains were little changed, with serious ratings for confounding (D1) and participant selection (D2) distributed across full publications and abstracts alike.

**Table 2:** Baseline Characteristics for RARP studies

Paper	Age		BMI		Pre-op PSA (ng/ mL)	
	Comparator	da Vinci^®^	Comparator	da Vinci^®^	Comparator	da Vinci^®^
Ragavan et al. (Hugo™)	68 (66–72)	68 (65–73)	24.6 (22.65–26.63)	25.14 (23.1-27.05)	12.39 (8.8–27)	22 (7.38-42)
Antonelli et al. (Hugo™)	65.9 ± 5.9	66.4 ± 5.5	25.4 (24.5–27.8)	27 (24.5–29.7)	7.7 (5.9–11.0)	5.9 (4.8–8.7)
Bravi et al. (Hugo™)	65 (60–70)	66 (61–71)	26 (24–29)	27 (25–30)	8.0 (5.7–11.1)	7.6 (5.1–11.3)
Brime Menendez et al. 2024 (Hugo™)	65.81 ± 8.1	66.33 ± 7.06	26.20 ± 3.98	25.40 ± 4.15	6.41 ± 1.9	6.88 ± 2.76
Gandi et al. (Hugo™)	68 (62.5–72)	67 (62–71)	26 (24.25–28)	27.04 (25–28.38)	7.7 (5.44-10)	8.0 (5.9-11.65)
Olsen et al. (Hugo™)	66 (63–73)	59 (58–62)	25.5 (23.7–27.5)	26.1 (24.7–26.7)	-	-
Ou et al. (Hugo™) ^‡^	66.50 (10.0)	67.50 (11.5)	25.79 (4.26)	26.00 (4.34)	8.81 (7.66)	9.46 (9.53)
Sultani Tehrani et al. (Hugo™)	61	61	26.9	26.8	9.6	6.9
Bianchi et al. (Hugo™)	-	-	-	-	-	-
Ko et al. (Hugo™)	67.5 ± 4.0	67.4 ± 3.1	26.1 ± 4.7	25.9 ± 4.0	10.2 ± 6.6	10.1 ± 5.9
Morizane et al. (Hugo™)	69.5 (67.5–73)	72 (67–74)	23 (22.9–26)	23.8 (21.2–26.1)	8.9 (6.8–11)	9.6 (6.5–13.7)
Piazza et al. (Hugo™)	-	-	27	28	-	-
Brime Menendez et al. 2026 (Hugo™)	64.76 ± 8.45	65.64 ± 7.32	-	-	7.91 ± 5.59	9.2 ± 5.79
Antonelli et al. (Versius^®^)	66.7 ± 6.3	66.4 ± 5.5	25 (23.0-28.4)	27 (24.5–29.7)	6.4 (5.0–9.0)	5.9 (4.8–8.7)
Dibitetto et al. (Versius^®^)	66 (61–72)	65 (57–74)	27 (23–32)	27 (23–31)	7.8 (5.2–10.4)	8.1 (5.1–12.7)

Values are presented as reported by the original studies: mean ± SD or median (Q1–Q3), unless otherwise indicated. ‡ Values reported as median (interquartile range width)

**Table 3:** Peri-operative Outcomes for RARP studies

Paper (Comparator)	Operative Time (min)		Estimated Blood Loss (mL)		Length of Stay (days)		Intra-operative Complications, *n* (%)		Major Complications (Clavien-Dindo ≥ 3)	
	Comparator	da Vinci^®^	Comparator	da Vinci^®^	Comparator	da Vinci^®^	Comparator	da Vinci^®^	Comparator	da Vinci^®^
Ragavan et al. (Hugo™)	195 (180–240)	210 (210–240)	-	-	1 (1–2)	1 (1–2)	0 (0%)	0 (0%)	-	-
Antonelli et al. (Hugo™)	-	-	300 (150–400)	200 (150–300)	4 (4–5)	4 (4–5)	3 (6%)	2 (4%)	2 (4%)	1 (2%)
Bravi et al. (Hugo™)	180 (150–200)	165 (130–200)	400 (250–500)	350 (200–500)	3 (3–4)	3 (2–4)	-	-	-	-
Brime Menendez et al. 2024 (Hugo™)	138.95 ± 45.51	145.34 ± 43.08	251.47 ± 76.98	328.27 ± 56.87	2.22 ± 0.42	2.12 ± 0.33	0 (0%)	0 (0%)	-	-
Gandi et al. (Hugo™)	170 (147.5–195.5)	166 (145–202.5)	100 (100–150)	100 (100–150)	4 (4–5)	4 (4–5)	-	-	-	-
Olsen et al. (Hugo™)	-	-	300 (150–400)	200 (100–350)	1 (1)	1 (1–2)	0 (0%)	0 (0%)	1 (5.3%)	0 (0%)
Ou et al. (Hugo™)	-	-	187.50	200.00	-	-	0 (0%)	0 (0%)	0 (0%)	0 (0%)
Sultani Tehrani et al. (Hugo™)	-	-	100	150	-	-	-	-	2 (6%)	0 (0%)
Bianchi et al. (Hugo™)	-	-	-	-	3	3	-	-	-	-
Ko et al. (Hugo™)	-	-	175 (100–200)	200 (163–463)	3 (2-3.8)	3 (2-3.8)	0 (0%)	0 (0%)	0 (0%)	0 (0%)
Morizane et al. (Hugo™)	278.5 (233.5–316)	348 (295–389)	162.5 (50-317.5)	200 (100–300)	9.5 (9–11)	10 (10–11)	0 (0%)	2 (2.5%)	-	-
Piazza et al. (Hugo™)	250 (220–290)	212 (137–380)	150	150	3	3	-	-	-	-
Brime Menendez et al. 2026 (Hugo™)	-	-	326 ± 205.69	302 ± 171.08	2.04 ± 0.35	2.04 ± 0.2	-	-	0 (0%)	0 (0%)
Antonelli et al. (Versius^®^)	-	-	250 (150–525)	200 (150–300)	-	-	3 (6%)	2 (4%)	-	-
Dibitetto et al. (Versius^®^)	170 (158–202)	96 (88–109)	350 (100–600)	250 (70–550)	3 (2–6)	3 (2–5)	-	-	4 (7.5%)	2 (4%)

**Table 4:** Oncological and Functional Outcomes for RARP studies

Paper	Positive Surgical Margins, *n* (%)		Continence Rate at 3 months, *n* (%)		Erectile Function at 3 months, *n* (%)	
	Comparator	da Vinci^®^	Comparator	da Vinci^®^	Comparator	da Vinci^®^
Ragavan et al. (Hugo™)	4 (23.53%)	4 (23.53%)	17 (100%)	17 (100%)	-	-
Antonelli et al. (Hugo™)	11 (22%)	13 (26%)	-	-	-	-
Bravi et al. (Hugo™)	20 (12%)	55 (15%)	133 (81%)	297 (79%)	-	-
Brime Menendez et al. 2024 (Hugo™)	15 (20%)	8 (10.67%)	-	-	-	-
Gandi et al. (Hugo™)	22 (22.2%)	25 (25.3%)	74 (74.7%)	73 (73.7%)	-	-
Olsen et al. (Hugo™)	7 (37%)	3 (27%)	11 (58%)	9 (82%)	10 (53%)	5 (45%)
Ou et al. (Hugo™)	6 (20%)	9 (30%)	23 (76.7%)	16 (53.3%)	22 (73.3%)	16 (53.3%)
Sultani Tehrani et al. (Hugo™)	3 (9%)	3 (9%)	-	-	-	-
Bianchi et al. (Hugo™)	-	-	22 (84%)	23 (88%)	-	-
Ko et al. (Hugo™)	6 (30%)	8 (40%)	12 (60%)	10 (50%)	1(5%)	0 (0%)
Morizane et al. (Hugo™)	6 (37.5%)	24 (29.6%)	-	-	-	-
Piazza et al. (Hugo™)	31 (29%)	61 (30%)	-	-	-	-
Brime Menendez et al. 2026 (Hugo™)	8 (32%)	7 (28%)	-	-	-	-
Antonelli et al. (Versius^®^)	-	-	-	-	-	-
Dibitetto et al. (Versius^®^)	9 (17%)	6 (11%)	29 (55%)	39 (74%)	-	-

**Table 5:** Baseline Characteristics for RAPN studies

Paper	Age		BMI		Tumour Size (mm)		*R*.E.*N*.A.L Score	
	Comparator	da Vinci^®^	Comparator	da Vinci^®^	Comparator	da Vinci^®^	Comparator	da Vinci^®^
Hevia Palacios et al. (Hugo™) ^‡^	66 (17)	59 (14)	26.6 (7.7)	27.9 (5.5)	45 (22)	40 (17)	6	6.5
García Rojo et al. (Hugo™)	61.48 ± 9.71	63.56 ± 9.31	27.9 ± 3.89	26.5 ± 4.11	35 ± 21.3	32.9 ± 11.66	5.76 ± 2.81	5.6 ± 2.12
Davies et al. (Hugo™)	53.4	55.8	28.4	28.3	-	-	-	-
Prata et al. (Hugo™)	63 (56–72)	63 (54.5–68)	27.4 (24.8–29.9)	26.6 (23.7–29.5)	34 (25–50)	35 (20–45)	-	-
Hussein et al. (Versius^®^)	45 (26–50)	50 (34–58)	-	-	-	-	-	-

Values are presented as reported by the original studies: mean ± SD or median (Q1–Q3), unless otherwise indicated. ‡ Values reported as median (interquartile range width)

**Table 6:** Peri-operative Outcomes for RAPN studies

Paper	Operative Time (min)		Warm Ischaemia Time (min)		Estimated Blood Loss (mL)		Intra-operative Complications, *n* (%)		Major Complications (Clavien-Dindo ≥ 3)	
	Comparator	da Vinci^®^	Comparator	da Vinci^®^	Comparator	da Vinci^®^	Comparator	da Vinci^®^	Comparator	da Vinci^®^
Hevia Palacios et al. (Hugo™)	-	-	19	24	-	-	0	0	0	1 (5.6%)
García Rojo et al. (Hugo™)	-	-	9.92 ± 11.94	14.38 ± 15.61	142.4 ± 85.25	233.8 ± 265.75	0	0	1(4%)	1(4%)
Davies et al. (Hugo™)	-	-	19.2	17.8	102	183	0	47 (13.7%)	-	-
Prata et al. (Hugo™)	112.5 (68.5–160)	67.5 (55–84)	-	-	271.3 (179.7–318.9)	172.5 (117.2–269.6)	-	-	1 (2%)	0
Hussein et al. (Versius^®^)	170 (140–180)	185 (180–258)	-	-	450 (150–500)	400 (275–1050)	-	-	-	-

Values are presented as reported by the original studies: mean ± SD or median (Q1–Q3), unless otherwise indicated

**Table 7:** Oncological and Functional Outcomes for RAPN studies

Paper	Positive Surgical Margins, *n* (%)		Change in eGFR at 3 months, (%)		Trifecta Rate, *n* (%)	
	Comparator	da Vinci^®^	Comparator	da Vinci^®^	Comparator	da Vinci^®^
Hevia Palacios et al. (Hugo™)	3 (17.6%)	2 (11.1%)	-	-	-	-
García Rojo et al. (Hugo™)	2 (8%)	1 (4%)	-	-	21 (84%)	22 (88%)
Davies et al. (Hugo™)	0 (0%)	0 (0%)	-4.2	-6.8	-	-
Prata et al. (Hugo™)	3 (5.8%)	0 (0%)	+0.5 (-7.4 - 10.9)	-7.3 (-16.4 - 0.5)	46 (89%)	47 (90%)
Hussein et al. (Versius^®^)	-	-	-	-	-	-

## Discussion

### Summary of findings

Across all included procedures, no consistent differences in peri-operative, oncological and early functional outcomes were identified between Hugo™ RAS or Versius^®^ and da Vinci^®^. This should be interpreted as an absence of evidence of a difference rather than as evidence of comparable effectiveness. In RARP, EBL, LOS and PSM showed overlapping ranges with no consistent direction of effect across studies, with one study reporting a significantly higher PSM rate in the Hugo™ arm [40]. Urinary continence at three months was similar in the larger datasets, with the smallest cohorts showing wider outcome ranges. Erectile function data remained too heterogeneous for meaningful comparison, with one study reporting significantly lower mean IIEF-5 scores in the Hugo™ arm [36]. In RAPN, WIT and trifecta achievement were closely similar in magnitude across reporting studies, although the number of studies reporting trifecta was small and the updated Prata analysis reported a difference approaching significance (*p* = 0.055) [27]. The single eligible pyeloplasty study reported identical median operative times and overlapping ranges for EBL and LOS, although with only 9 and 25 cases respectively it provides minimal comparative information [22]. No comparative cystectomy data were identified, representing an important gap in the current evidence base.

These findings are concordant with a recent, comprehensive systematic review of novel multiport platforms. Reitano et al. meta-analysed 74 studies and 5,487 patients across nine novel multiport platforms and found no significant differences from da Vinci^®^ in operative time, EBL, complications, PSM, three-month continence or trifecta achievement [18]. Docking time was the only outcome they could analyse for Hugo™ RAS specifically, being reported solely in Hugo studies, and was significantly longer (MD 6.2 min, 95% CI 4.2–8.2) - mirroring the setup and docking pattern seen across our RARP cohort. They also identified no comparative cystectomy data, with novel-platform cystectomies having been described only in case reports. Their remaining pooled estimates nonetheless combined nine platforms into a single comparator arm, so platform-specific effects were otherwise not recoverable. The authors also reported substantial heterogeneity, high risk of bias in all but three studies, and small sample sizes. The limitations support cautious interpretation rather than a conclusion of equivalence.

### Generalisability and learning curve

All studies included in this review came from high-volume specialist centres and were performed by surgeons with extensive prior robotic experience. This is to be expected in the first few years following introduction of a novel platform. Prior robotic experience was explicitly reported in seventeen of nineteen studies and was uniformly high, with stated da Vinci^®^ caseloads ranging from 50 to over 1000 RARPs, and six studies additionally described dedicated manufacturer or academy training before the novel platform was introduced [24, 28, 37, 39, 40, 41]. Even under these conditions, there is a significant learning curve when adopting new technology. Bianchi et al. showed significantly longer operative times persisting for the first 20 cases [33].

The extent to which comparisons were confounded by centre, surgeon and study period varied. Nine studies explicitly stated that the same surgeons operated on both arms, which more plausibly isolates platform effects. Two studies explicitly did not: Prata et al. compared platforms used in different centres by different surgeons and acknowledged that observed differences may partly reflect variation in surgeon experience [26], while Morizane et al. reported that only expert surgeons held certification for the newer systems at introduction, so scheduling preferentially allocated them to those platforms - the authors’ own explanation for their paradoxical finding of shorter Hugo™ RAS operative times [35]. Only one study excluded early learning-curve cases, Gandi et al. starting Hugo™ RAS inclusion from the eleventh case [28], meaning most studies compared a platform in its introductory phase against an established platform - a design that systematically disadvantages the newer system. Reported learning-curve breakpoints ranged from 17 to 30 cases [23, 24, 33], so studies contributing fewer than approximately 30 cases per arm may capture predominantly early learning-phase performance.

Furthermore, functional recovery following RARP is known to continue improving for up to twelve months postoperatively [43], meaning three-month outcome data across included studies are unlikely to capture the full functional trajectory for either platform. This review highlights a clear research gap: outcomes and learning-curve trajectories in centres without an established robotic programme remain uncertain.

### Health system considerations and market dynamics

Despite early comparative results showing no consistent outcome differences, adoption of these platforms remains markedly uneven globally. Cannoletta et al. mapped uro-oncological experience across ten platforms and identified studies from 42 countries for da Vinci^®^ compared with 12 for Hugo™ RAS, with the remaining systems collectively represented in 13 countries and almost no published evidence from Africa or South America. They attribute this distribution principally to cost rather than to outcome data [48]. They further note that the most robust Versius^®^ evidence has come from the United Kingdom and Italy, consistent with the geographical concentration of the studies used in our review. The comparative evidence synthesised here therefore comes from a small, well-resourced subset of the centres and countries in which these platforms are ultimately intended to expand access.

Several practical barriers to adoption of novel platforms have been proposed, including retraining theatre teams, early learning curve costs and the institutional risk of moving away from an established platform with two decades of safety data. These factors may influence adoption independent of clinical outcomes. Notably, the Hussein et al. cohort from Sindh Institute of Urology and Transplantation (SIUT) [22] had transitioned to Versius^®^ only after what the authors described as a reduction in Intuitive Surgical’s programme support, illustrating that access to robotic systems and institutional or commercial factors may also influence platform selection. Two cost analyses have suggested that Hugo™ RAS may offer lower direct costs than the da Vinci^®^ [44, 45], raising the possibility that lower direct costs could facilitate adoption in some healthcare systems. More importantly, the entry of lower cost platforms into the market creates genuine competition with the potential to drive down costs across all robotic systems and help accelerate innovation – an outcome which would benefit patients and healthcare systems, regardless of which platform ultimately prevails.

### Platform-specific performance & technical issues

Beyond comparing outcomes, several studies highlighted relevant differences in technical performance which are not captured by the headline data. Setup and docking times were consistently longer with Hugo™ RAS, even in experienced hands, reflecting the demands of its modular arm configuration. The Hugo™ RAS series also consistently reported higher rates of intra-operative device malfunction compared to the da Vinci^®^, including arm calibration errors, instrument failures, and console alerts. These events were not reported to have resulted in clinical harm but may have disrupted operative workflow in ways not captured by conventional peri-operative endpoints. Antonelli et al. recorded 20 malfunctioning events versus 4 with da Vinci^®^, half of which required over five minutes to resolve [24]. Piazza et al. also reported Hugo™ RAS technical failure rates of 14% versus 0.5% (*p* < 0.001) [38]. The higher PSM rate reported by Brime Menendez et al. 2024 [40] was similarly attributed to a hardware limitation with the grasping forceps. A software update in March 2024 by Medtronic addressed many reported technical issues, including alarm and arm collision errors, underscoring that these platforms are still being actively refined [46]. Neither platform is a straightforward drop-in replacement for da Vinci^®^, and both require dedicated theatre team training and realistic expectations during transition. It is also worth noting that the open console design shared by both Hugo™ and Versius^®^ may offer potential advantages for surgical education and team observation by allowing additional team members to observe the surgeon’s hand movements in real time; however these potential benefits have not been formally evaluated in comparative studies.

### Strengths, limitations & future directions

This review was prospectively registered on PROSPERO, conducted in accordance with PRISMA guidelines, and involved independent dual-reviewer screening across five databases. Its principal contribution is not the finding that outcomes appear broadly similar, (consistent with existing syntheses), but rather the platform-stratified and procedure-specific structure of that finding: that the Hugo™ RAS evidence base is substantially larger than the Versius^®^ one and that the consistent differences identified concern setup, docking and device reliability rather than clinical outcomes.

The limitations of the evidence base should be understood in the context of where these novel platforms sit in their adoption trajectory. As first-generation systems, Hugo™ and Versius^®^ are inherently at an early, uncertain stage of implementation. The absence of randomised designs and long-term follow-up is consistent with this stage of adoption, but nonetheless substantially limits the conclusions that can be drawn. Sample sizes ranged from less than 20 to a few hundred patients per arm. Studies of this size have limited power to detect differences in low-frequency outcomes such as major complications or PSM, and the repeated finding of non-significance across the evidence base should not be taken as a conclusion of similarity. Outcome definitions also varied across studies, as detailed in Sects.  3.4.2 and 3.5.2, and publication bias towards positive results from academic centres remains a concern.

The coverage of the evidence base is also uneven. There was marked asymmetry between platforms: 17 studies contributed Hugo™ comparisons and only 3 contributed Versius^®^ comparisons (with one study contributing to both). The only eligible Versius^®^ RAPN study reported outcomes for multiple procedures rather than for RAPN specifically, further limiting conclusions that can be drawn about the Versius^®^ performance in this context. The absence of any comparative cystectomy data represents a significant evidence gap. Our conclusions are also restricted to the four index procedures specified in the protocol and cannot be extrapolated to other urological procedures performed with these platforms, such as adrenalectomy, nephroureterectomy or simple prostatectomy, for which comparative data were not sought. Future research should prioritise prospective multicentre studies with standardised outcome reporting, longer oncological follow-up and cost-effectiveness analyses to inform commissioning decisions.

## Conclusions

This systematic review did not identify consistent differences in peri-operative, oncological or early functional outcomes between the Hugo™ RAS or Versius^®^ platforms and the da Vinci^®^ system across RARP, RAPN and pyeloplasty in experienced centres. These findings should not be read as evidence that the platforms are of comparable effectiveness - the available studies were predominantly small, non-randomised, and conducted in high-volume specialist centres, and were not designed or powered to detect clinically meaningful differences or to demonstrate equivalence. Setup and docking times were consistently longer with Hugo™ RAS, while higher rates of intra-operative device malfunction were reported across several series. The absence of comparative data for radical cystectomy, the limited Versius^®^ evidence base, and the lack of long-term oncological and functional outcomes represent important gaps. Prospective multicentre studies with standardised outcome definitions, longer follow-up and inclusion of centres at different stages of robotic experience are required to further define the clinical, technical and economic role of these platforms in urological surgery.

## Data Availability

All data analysed in this review were extracted from previously published studies, which are cited in the reference list. The extracted outcome data supporting the findings are presented in Tables 1, 2, 3, 4, 5, 6 and 7 of this article. The complete extraction dataset is available from the corresponding author on request. The review protocol was prospectively registered with PROSPERO (CRD420261319741).

## Appendix

**Appendix A.** Search strategy
